# Video Grading of Bile Duct Anastomoses During Robotic Pancreatoduodenectomy Predicts Bile Leak and Identifies the Learning Curve: A Multicenter Study

**DOI:** 10.1097/SLA.0000000000006673

**Published:** 2025-02-17

**Authors:** Maurice J.W. Zwart, Bram L.J. van den Broek, Sabrina L.M. Zwetsloot, Olivier R. Busch, T.C. Khé Tran, Misha D. Luyer, Jennifer Schreinemakers, Jan H. Wijsman, George P. van der Schelling, Ignace H.J.T. de Hingh, J. Sven D. Mieog, Bert A. Bonsing, Kosei Takagi, Roeland F. de Wilde, Herbert J. Zeh, Amer H. Zureikat, Melissa E. Hogg, Bas Groot Koerkamp, Marc G. Besselink

**Affiliations:** * Department of Surgery, Amsterdam UMC, University of Amsterdam, Amsterdam, The Netherlands; † Cancer Center Amsterdam, The Netherlands; ‡ Department of Surgery, Erasmus University Medical Center, Rotterdam, The Netherlands; § Department of Surgery, Catharina Medical Center, Eindhoven, The Netherlands; ∥ Department of Surgery, Amphia Ziekenhuis, Breda, The Netherlands; ¶ Department of Surgery, Leiden University Medical Center, Leiden, The Netherlands; # Department of Surgery, University of Texas Southwestern Medical Center, Dallas, TX; ** Department of Surgery, Division of GI Surgical Oncology, University of Pittsburgh Medical Center, Pittsburgh, PA; †† Department of Surgery, NorthShore University HealthSystem, Evanston, IL

**Keywords:** bile leak, hepaticojejunostomy, OSATS, robotic pancreatoduodenectomy

## Abstract

**Objective:**

To determine whether video grading using objective structured assessment of technical skills (OSATS) could estimate the risk of postoperative bile leak (BL) after robotic pancreatoduodenectomy (RPD) and to identify a learning curve effect.

**Background:**

The hepaticojejunostomy (HJ) BL rate after RPD is rather high, with 10%, and may be improved by structured training and skills. Robotic HJ, therefore, requires confirmation of adequate performance. Grading of surgical performance during HJ could be used in competency-based surgical training.

**Methods:**

Post hoc analysis of patients included the Dutch LAELAPS-3 RPD training program in 6 centers. Technical performance during robotic HJ was graded by 2 blinded graders using OSATS (attainable scores: 6 to 30). Primary outcome was grade B/C BL, according to the International Study Group of Liver Surgery. Logistic regression determined the performance cutoff, and cumulative sum analysis identified the learning curve.

**Results:**

Videos from robotic HJ in 259 patients were included with a 6.9% rate of grade B/C BL (n = 18/259). The median OSATS for the HJ was 25.0 (22 to 27), with an OSATS score >21 associated with a reduced risk of BL. The rate of BL was 5.1% in patients for OSATS >21 and 12.5% for OSATS ≤21, amounting to a relative reduction of 59.2% and an absolute reduction of 7.4% [8/64 vs 10/195, odds ratio (OR): 0.378, *P* = 0.013]. These findings remained similar when only including grade C BL (OR: 0.076, *P* = 0.004. On multivariable analysis for grade B/C BL, the only significant predictive factor was OSATS >21 (OR: 0.273, *P* = 0.025). Stabilization of the cumulative sum learning curve for grade B/C BL was reached at 19 RPD procedures, and after 44 procedures, the learning curve showed a continuous downward trend. The rate of grade B/C BL was significantly lower beyond 19 RPD: 5.6% versus 8.6% (8/143 vs 10/116, OR: 0.710, *P* = 0.040).

**Conclusions:**

The risk of postoperative BL after RPD is strongly associated with surgical performance during robotic HJ as objectified using OSATS. This approach can be used for rapid assessment of the learning curve and competency-based surgical training, aiming for a safe implementation of RPD.

In experienced centers, the mortality rate after pancreatoduodenectomy (PD) is now below 3%.^1–3^ However, the morbidity rate after PD remains considerable and includes postoperative pancreatic fistula (POPF), delayed gastric emptying, and bile leak (BL). Most commonly, BL is related to hepaticojejunostomy (HJ) leakage, which is also one of the less-studied complications after PD. Multicenter studies report a rate of grade B/C BL after PD of 3% to 10%,^4–6^ which leads to prolonged hospital stay, morbidity,^7^ interventional procedures, and even mortality.^8^

Several patient-related risk factors associated with increased biliary complications have been identified, such as preoperative stent use and ductal diameter.^7,9^ Technical risk factors further contribute to increased risk of biliary complication,^10^ as well as lower volume centers.^11^ In recent years, the use of robot-assisted PD (RPD) has been increasing, which provides an excellent opportunity for structured assessment of surgical videos during the learning curve. Video scoring enables objective assessment of surgical performance for clinical outcomes.^12,13^ However, it is currently unclear how surgical performance affects the risk of BL after RPD.

Two earlier studies demonstrated the value of video grading of the surgeon’s technical performance of pancreatic anastomoses during RPD to predict the risk of grade B/C POPF.^14,15^ One study identified technical factors predictive of biliary complications in 241 RPDs.^10^ The objective structured assessment of technical skill (OSATS) system is widely used in assessing the skill of surgical trainees,^16^ and was used for predicting complications after bariatric procedures and RPD.^14,15,17^ Yet, data on this concept in multicenter studies regarding BL in RPD are lacking.^14^ This is of interest, more than POPF, as BL could be largely related to surgical skills. The 10% rate of BL reported after RPD could, therefore, be related to a learning curve effect.^18,19^

The aim of the current study was to determine whether video grading using OSATS of robotic HJ during RPD could identify a learning curve and estimate the risk of postoperative BL after RPD.

## METHODS

### Study Design

This was a substudy within the prospectively observational multicenter LAELAPS-3 trial.^18,19^ Videos were graded following the OSATS score by 2 blinded graders (M.J.W.Z. and B.L.J.v.d.B.). The videos were recorded in 5 centers in the Netherlands between 2017 and 2021. The outcomes are associated with the prospectively maintained database of the LAELAPS-3 study including all consecutive patients who underwent robotic PD between 2017 and 2021. This substudy of the LAELAPS-3 trial was approved by the Institutional Review Board of the participating centers (NTR NL8073).

### Eligibility

Patients who underwent RPD for all indications were eligible. Patients were excluded when their video did not include a complete HJ, or when the procedure was converted to the open approach before the HJ. The HJ comprised the following steps in the video: (1) setting up bowel, (2) jejunal enterotomy, (3) suturing, (4) determining the length of jejunum between the HJ and gastrojejunostomy, and (5) checking the anastomosis for leakage and placing additional sutures (optional). The surgeons participated as part of the LAELAPS-3 RPD training program. As such, they are all considered at the beginning of their learning curve for RPD and worked at a center capable of performing 20 RPDs annually (around ~50 open PDs annually). Fellows performing anastomoses were always directly supervised by the attending surgeon. Residents did not perform anastomoses. These surgeons did not perform laparoscopic PDs during the study participation; the surgeons with experience in laparoscopic surgery performed 30 to 50 laparoscopic PDs each. All surgeons had performed >100 open PD procedures.

### Hepaticojejunostomy Technique

Dissection of the hepatic pedicle was performed with the robotic hook, bipolar forceps and/or assistance with a laparoscopic sealing device. In case of bleeding from the bile duct vessels, suture ligation with 5-0 PDS, and the use of vessel sealing and/or electrocautery was limited to a minimum. Transection of the bile duct was performed above or below the level of cystic duct insertion using the shears (in some instances electrocautery was used). The anastomosis was completed with two 4-0 barbed sutures (V-loc by Covidien, Medtronic or Stratafix by Ethicon, Johnson & Johnson) in case of a wide thick-walled bile duct or with interrupted 5-0 monofilament sutures (PDS by Ethicon, Johnson & Johnson or Maxon by Covidien, Medtronic) in case of a narrow thin-walled bile duct, both with an atraumatic needle. Different needle drivers were used, such as the diamond-coated micro-forceps, large needle drivers, either with or without suture-cutting function. In the majority of procedures, the length between the HJ and gastrojejunostomy was measured using a 50 to 60 cm thread, and a 5x5 cm gauze was placed around the HJ after complication and inspected again after 5 to 10 minutes to indicate BL. Approximately 20 minutes before starting with the HJ, surgeons in some centers administered ICG-green to detect bile leakage during the HJ. In case of BL, the HJ was revised. One center used tissue glue at the beginning of their experience as part of a center-specific protocol, and one center employed tissue glue in case of a soft pancreas to protect against eventual POPF.

### Outcomes and Definitions

Patient demographics variables included: age, body mass index (BMI), and sex. Surgeon demographic variables included type of center, first surgeon (including years of overall, minimally invasive (MI), and laparoscopic PD experience), and high volume or not (cutoff: annual surgeon volume of >20 PDs). Intraoperative variables included video review data collection: time to complete the HJ anastomosis, suturing time, bile duct diameter based on the millimeter indicator on the needle driver, aspect of the bile duct (normal or either fibrosed or inflamed), use of a temporary bulldog clamp to prevent leakage of bile after transection of the bile duct, stent use, tissue glue, teres ligament patch (ie, the teres hepatis portion of the falciform ligament), additional segments that required a bilio-enteric anastomosis, proximity of the HJ anastomosis to the hilar plate, suturing technique (interrupted vs continuous), type of suture material.

Postoperative variables were collected during the entire hospital stay and, in case of earlier discharge, up to 30 days postoperatively. Clinically relevant BL is defined as the need for radiologic (grade B) or operative intervention (grade C) resulting from biliary collections or bile peritonitis, according to the International Study Group of Liver Surgery.^20^

The primary outcome was grade B/C BL and its association with OSATS performance cut-off. Secondary outcomes were the learning curve analysis and the intergrader variability of the OSATS score. Other outcomes were predictive value of other risk factors for BL (including previous surgical experience), and intergrader variability.

### Grading

The graders scored the videos using the OSATS system, which comprises 6 variables: gentleness, time and motion, instrument handling, flow of operation, tissue exposure, and overall technical skill (for more information, see Table 2). Total OSATS score was calculated by adding up the scores attributed to each of the 6 variables, attainable scores were 6 to 30. Two graders independently graded HJs blinded for patient outcomes, participating centers, and the operating surgeon. One experienced grader (M.J.W.Z.) was trained in the UPMC (by M.E.H., A.H.Z., and H.J.Z.). The second grader was an intermediately experienced grader (B.A.B.) who was trained by an experienced grader (M.J.W.Z.) and who had performed grading in the LAELAPS-3 pancreatico-jejunostomy grading study.^15^ The second grader served as an internal validation of a random sample of 65 HJs, which were selected with a random selection computer program on SPSS.

### Statistical Analysis

Data were analyzed using IBM SPSS statistics for Windows version 28 (IBM Corp). Continuous data that are normally distributed are presented as mean and SD. Continuous data not normally distributed are presented as a median and interquartile range (IQR) or 95% CI, with Alpha set to 0.8, or 80%. Binary, nominal, and ordinal (or: categorical) data are presented as frequencies and percentages. Outcomes are compared and assessed for significance (2-tailed *P* value of <0.05) with the Student *t* test for normally distributed data, χ^2^ test for frequencies in one or more categories (accounting for ordinal categories), and Mann-Whitney *U* test for non-normally distributed data. The predictability of OSATS was tested using logistic regression. The effect of OSATS on BL and the effect of surgeon characteristics on OSATS were assessed through univariable and multivariable analysis. For the intergrader analysis, the Spearman correlation was used. Learning curve analyses were performed to investigate OSATS correlation to the consecutive case number of each surgeon. Cumulative sum (CUSUM) analysis was used to assess a learning curve turning point. The CUSUM curve represents the aggregate of individual learning curves of surgeons in the study, with each surgeon’s consecutive case number contributing to the total. Some surgeons performed significantly more procedures, thus extending the CUSUM curve beyond the average. The minimal OSATS score for minimizing the risk of BL was determined through threshold analysis with logistic regression, and the cutoff with the biggest impact on BL was chosen. A sensitivity analysis excluding concomitant POPF cases was performed, aiming to exclude the possible influence of leakage of bile through the pancreatic fistula (“dirty leak”).

## RESULTS

Of the 367 patients after RPD assessed, 259 could be included. Reasons for exclusion were: conversion to open procedure (n = 11), failure to record or corrupted video (n = 90), and incomplete video (n = 7; Fig. 1). A total of 259 videos were analyzed. Grade B/C BL occurred in 18/259 patients (6.9%), of which 5/259 grade C (1.9%).

**FIGURE 1:**
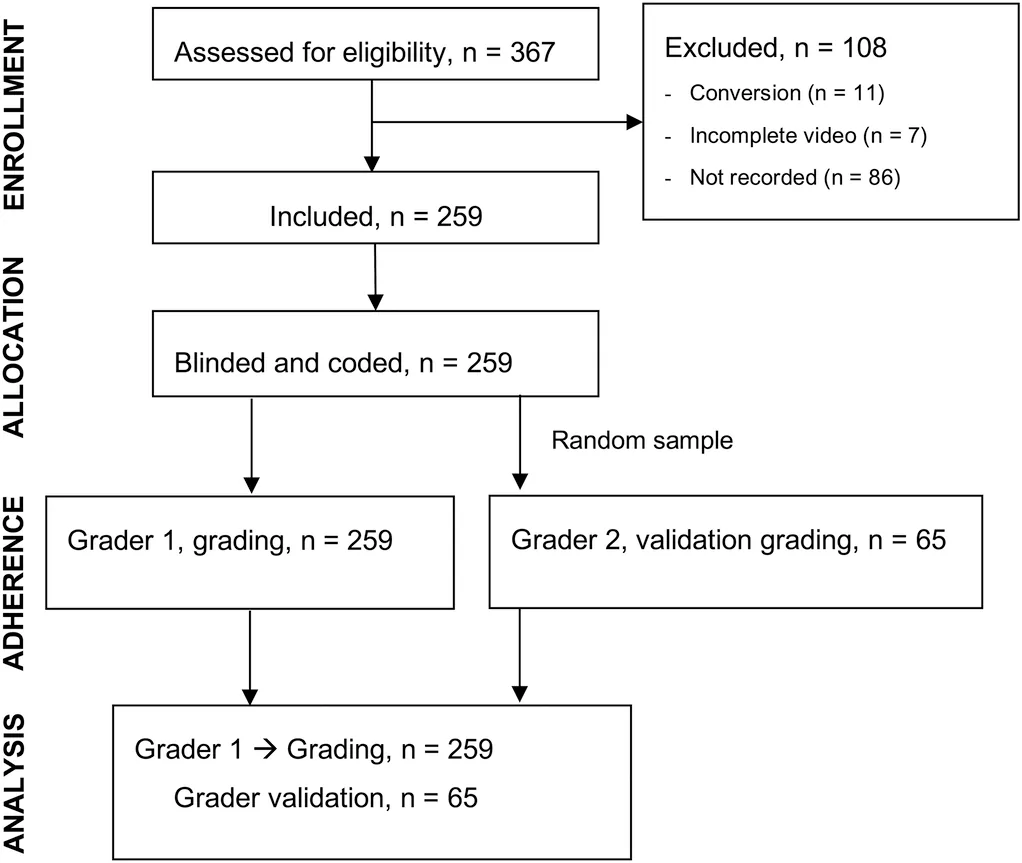
Flowchart of enrollment.

### Patient Characteristics

The mean age of included patients was 67 years (±10), with slightly more males (n = 141/259, 54.4%). The median BMI was 25 kg/m^2^ (IQR: 23 to 28). Criteria for the American Society of Anesthesiologists ≥III were met by 83/258 patients (32.0%). Adenocarcinoma was diagnosed in 187/259 patients (72.2%). See Table 1 for all available baseline demographics.

**TABLE 1:** Baseline Characteristics, Stratified for OSATS Score Cutoff >21

Baseline characteristics	Total (n = 259)	OSATS >21 (n = 195)	OSATS ≤21 (n = 64)	*P*
Age (yr), mean±SD	67±10	66±10	67±11	0.703
BMI (kg/m^2^), median (IQR)	25 (23 to 28)	25 (23 to 27)	25 (23 to 28)	0.497
ASA physical status ≥III, n (%)	83 (32.0)	62 (31.8)	21 (32.8)	0.657
Adenocarcinoma, n (%)	187 (72.2)	144 (73.8)	43 (67.2)	0.302
HJ characteristics				
Bile duct diameter (mm), median (IQR)	10 (7 to 15)	10 (7 to 15)	10 (7 to 15)	0.385
Bile duct aspect fibrosed aspect, n (%)	60 (23.3)	45 (23.1)	15 (23.4)	0.953
Additional segment reconstruction, n (%)	19 (7.3)	15 (7.7)	4 (6.3)	0.701
Distance to hilar plate, mm, median (IQR)	17 (10 to 25)	18 (10 to 25)	10 (6 to 24)	0.103
Transected with cautery, n (%)	105 (40.7)	91 (46.7)	14 (21.9)	0.001
Temporary bulldog clamp^*^, n (%)	74 (28.7)	58 (29.7)	16 (25.0)	0.466
HJ standing, n (%)	154 (59.5)	127 (65.1)	27 (42.2)	0.001
Use of barbed suture, n (%)	107 (41.3)	68 (34.9)	39 (60.9)	<0.001
Mitigating strategies, n (%)				
HJ revision	96 (37.1)	67 (34.4)	28 (43.8)	0.176
Stent	74 (28.6)	58 (29.7)	16 (25.0)	0.446
Tissue glue	24 (9.3)	23 (11.8)	1 (1.6)	0.014
Falciform patch	12 (4.6)	10 (5.1)	2 (3.1)	0.508
Surgeon characteristics				
University Medical Center, n (%)	221 (85.3)	176 (90.3)	45 (70.3)	<0.001
By attending surgeon, n (%)	191 (73.7)	137 (70.3)	54 (84.4)	0.026
Prior surgical experience (yr), median (IQR)	15 (7 to 16)	15 (7 to 16)	15 (11 to 17)	0.164
Prior MI surgical experience (yr), median (IQR)	4 (1 to 11)	4 (1 to 11)	9 (1 to 14)	0.029
By surgeon with LPD experience, n (%)	105 (40.5)	75 (38.5)	30 (46.9)	0.234
Surgeon annual volume >20 PDs, n (%)	226 (87.3)	179 (91.8)	47 (73.4)	<0.001
Procedure characteristic				
HJ time (min), median (IQR)	38 (28 to 48)	38 (28 to 46)	38 (26 to 52)	0.444
HJ suturing time (min), median (IQR)	28 (21 to 35)	28 (21 to 35)	30 (24 to 38)	0.698

^*^ Placement after transection of the bile duct.

ASA indicates American Society of Anesthesiologists Physical Status Classification System; BMI, Body mass index; HJ, Hepaticojejunostomy; IQR, interquartile range; y, years.

### Hepaticojejunostom Characteristics

The median bile duct diameter was 10 mm (IQR: 7 to 15). In 60/259 (23.2%) patients, the bile duct showed an inflamed and/or thickened aspect. In 19/259 patients (7.3%), the biliary tree had a separated segment that required additional reconstruction with a biliodigestive anastomosis. The median distance of the transected bile duct to the hilar plate was 17 mm (IQR: 9 to 25). The bile duct was transected using cautery in 105/259 patients (40.5%). In 74/259 (29.6%) patients a bulldog clamp was placed on the transected bile duct to prevent bile spill. In the majority of patients, the HJ was completed proximal to the cystic duct (n = 198/259, 76.4%), others were at the cystic duct (n = 16/259, 6.2%) and distal to the cystic duct (n = 45/259, 16.1%). The time to complete the HJ was 37.7 minutes (IQR: 27.7 to 48.0), of which 28.0 minutes (IQR: 21.3 to 35.3) of suturing. HJs were completed with single-standing sutures in the majority of patients (n = 154/259, 59.5%), and an internal bile duct stent was used in 74/259 patients (28.6%). Suture materials used were: 146/259 PDS (63.0%), 77/259 V-loc (29.7%), 30/259 Stratafix (11.6%), and 5/259 Maxon (1.9%). Tissue glue was used in 24/259 patients (9.3%) and a falciform patch in 12/259 patients (4.6%). Overall, 96/259 (37.1%) HJs were revised intraoperatively after the gauze leakage test revealed biliary leakage using one or more additional sutures.

### Surgeon Characteristics

HJs were performed by 9 surgeons from 5 centers, of which 3 were university medical centers. Median years of surgical experience was 15 (IQR: 7 to 16), of which 4 (IQR: 1 to 11) with MI surgery. The average experience was 40.7 RPD procedures. Two surgeons reported an annual case surgeon volume of RPD <20, and 7 surgeons reported this volume as ≥20. Three of the 9 surgeons had prior experience with laparoscopic PD.

### Objective Structured Assessment of Technical Skills and Grade B/C Bile Leak

The median OSATS for performing the robotic HJ was 25 (IQR: 22 to 27). An OSATS of 21 was associated with a significantly lower risk of grade B/C BL [odds ratio (OR: 0.378, *P* = 0.013], and similar for grade C BL (OR: 0.076, *P* = 0.004). A grade B/C BL occurred in 10/195 patients (5.1%) with OSATS >21 and in 8/64 patients (12.5%) with OSATS ≤21, amounting to a relative reduction of 59.2% (absolute reduction: 7.4%). For more details, see Table 2.

**TABLE 2:** OSATS and BL According to OSATS Score Cutoff >21

Grading	Total (n = 259)	OSATS >21 (n = 195)	OSATS ≤21 (n = 64)	*P*
Total OSATS score, mean (95% CI)	23.9 (23.4 to 24.5)	26.0 (25.7 to 26.3)	17.7 (17.1 to 18.3)	<0.001
Gentleness, mean (95% CI)	3.6 (3.4 to 3.7)	4.0 (3.8 to 4.1)	2.5 (2.2 to 2.7)	<0.001
Time and motion, mean (95% CI)	4.3 (4.2 to 4.4)	4.6 (4.5 to 4.6)	3.6 (3.4 to 3.8)	<0.001
Instrument handling, mean (95% CI)	4.3 (4.2 to 4.5)	4.7 (4.6 to 4.7)	3.4 (3.2 to 3.6)	<0.001
Flow of operation, mean (95% CI)	4.0 (3.9 to 4.1)	4.4 (3.2 to 4.5)	2.9 (2.6 to 3.1)	<0.001
Tissue exposure, mean (95% CI)	4.1 (4.0 to 4.3)	4.5 (4.4 to 4.6)	3.1 (2.9 to 3.4)	<0.001
Overall technical skill, mean (95% CI)	3.6 (3.5 to 3.7)	4.0 (3.9 to 4.1)	2.3 (2.1 to 2.5)	<0.001
BL				
CR (grade B/C), n (%)	18 (6.9)	10 (5.1)	8 (12.5)	0.013
Grade C, n (%)	5 (1.9)	1 (0.5)	4 (6.3)	0.004

Gentleness = gentle tissue handling that does not result in injury; time and motion = economy of motion, maximum efficiency; instrument handling = fluid use of instruments without awkwardness; flow of operation = smooth transitions from one part of the operation to another; tissue exposure = retraction that allows for good visualization and proper tissue alignment; overall technical skill = overall assessment of technical skill.

1/5 = deficient/traumatic; 2/5 = lacking/lacks finesse; 3/5 = average; 4/5 = skilled; 5/5 = master/flawless.

CR indicates clinically relevant.

The rates of BL were significantly lower for several other OSATS score cutoffs, albeit with lower ORs; as mentioned in the methods, the cutoff with the biggest impact on BL was chosen; for a visual overview, see Appendix 1 (Supplemental Digital Content 1, http://links.lww.com/SLA/F413). Furthermore, all individual OSATS elements and total scores showed an inverse association with grade C BL (gentleness, Rho −0.096, *P* = 0.123; time and motion, Rho −0.184, *P* = 0.003; instrument handling, Rho −0.103, *P* < 0.001; tissue exposure, Rho −0.137, *P* = 0.028; flow of operation, Rho −0.139, *P* = 0.025; summary score, Rho −0.144, *P* = 0.020; HJ total, Rho −0.162, *P* = 0.009). For a visual representation of the rate of BL along with increasing OSATS scores, see Figure 2.

**FIGURE 2:**
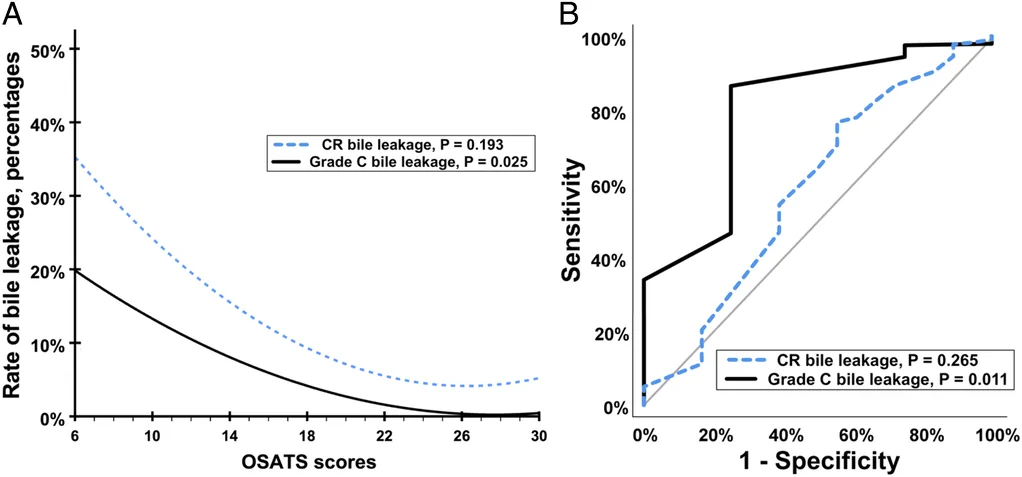
OSATS and the risk of grade B/C BL in robotic HJ. A, The regression analysis for curve estimation of risk of bile leakage (grade B = dashed line, grade C = solid line) along increasing OSATS scores. B, Receiver operating characteristic curve of OSATS as diagnostic for bile leakage (grade B = dashed line, grade C = solid line).

Baseline differences with multivariable analysis for risk of grade B/C BL showed that OSATS remained a consistent risk factor. There were no significant baseline differences between groups, that is, OSATS >21 (N = 193) versus OSATS ≤21 (N = 64), patients were similar in age, BMI, sex, and rate of adenocarcinoma. For HJ characteristics, there were several significant differences (for more information, see Table 1). However, on multivariable analysis for grade B/C BL, the only significant predictive factor was OSATS >21 (OR: 0.275; 95% CI: 0.088 to 0.849; *P* = 0.026; Table 3).

**TABLE 3:** Multivariable Analysis for Grade B/C BL

	Grade B/C BL			
	Univariable		Multivariable	
Baseline Characteristic	RR (95% CI)	*P*	OR (95% CI)	*P*
Increasing patient age (yr)	−0.05* (−0.18 to 0.07)	0.407	—	—
Patient BMI (kg/m2)	0.05* (−0.07 to 0.18)	0.397	—	—
sex (male patient)	1.10 (0.69 to 1.78)	0.750	—	—
Adenocarcinoma (yes)	0.48 (0.17 to 1.32)	0.147	—	—
HJ characteristics				
Increasing bile duct diameter (mm)	0.04* (−0.09 to 0.17)	0.320	—	—
Bile duct with fibrosed aspect	0.94 (0.30 to 2.97)	0.922	—	—
Additional segment	0.92 (0.89 to 0.96)	0.216	—	—
Increasing distance to hilar plate (mm)	−0.02* (−0.15 to 0.11)	0.759	—	—
Transected with cautery†	1.51 (0.58 to 3.94)	0.397	1.75 (0.47 to 6.47)	0.405
Temporary bull dog clamp‡	0.29 (0.07 to 1.31)	0.191	—	—
HJ standing sutures†	0.84 (0.32 to 2.21)	0.727	1.74 (0.26 to 11.80)	0.570
HJ with running barbed suture†	1.46 (0.56 to 3.81)	0.438	1.80 (0.26 to 12.50)	0.550
Mitigating strategies				
Revision of the anastomosis	1.39 (0.53 to 3.65)	0.504	—	—
Stent	1.65 (0.62 to 4.44)	0.315	—	—
Tissue glue†	2.10 (0.56 to 7.83)	0.262	2.15 (0.42 to 11.03)	0.359
Falciform patch/teres ligament	0.95 (0.92 to 0.98)	0.332	—	—
Surgeon characteristics				
Academic center†	1.41 (0.31 to 6.37)	0.658	0.28 (0.02 to 4.25)	0.356
Attending surgeon†	1.85 (0.52 to 6.59)	0.338	1.37 (0.23 to 8.16)	0.733
Increasing surgical experience (yr)	−0.01* (−0.12 to 0.13)	0.879	—	—
Increasing MI surgical experience (yr)†	−0.01* (−0.14 to 0.11)	0.805	0.99 (0.85 to 1.14)	0.862
Surgeon annual volume >20 PDs†	2.60 (0.34 to 20.24)	0.727	0.07 (<0.01 to 4.52)	0.214
Prior experience in LPD	1.19 (0.45 to 3.12)	0.642	—	—
OSATS performance cutoff				
Cutoff >21	0.38 (0.30 to 0.92)	0.013	0.27 (0.09 to 0.85)	0.025

*P* value (based on χ2).

^*^ Spearman Rho.

^†^ Included in multivariable analysis because of significant differences between OSATS groups.

^‡^ Placement after transection of the bile duct.

### Learning Curve and Grade B/C Bile Leak

CUSUM analysis was performed both for learning curve analysis (using OSATS scores) and for HJ operative time. For OSATS, a significant association with consecutive case numbers was observed at 0.270 (95% CI: 0.153 to 0.380). Stabilization of the CUSUM learning curve was reached at 19 robotic HJ procedures, and after 44 procedures, the learning curve showed a continuous downward trend (Fig. 3). The OSATS scores were significantly higher after both turning points: >19 procedures, with a median of 23 versus 26 points, *P* <0.001 and >44 procedures, median 23 versus 27 points, *P* <0.001. A lower rate of grade B/C BL was observed beyond 19 robotic HJs performed per surgeon: 10/116 (8.6%) versus 8/143 (5.6%), OR 0.710, relative reduction 34.9% (absolute reduction 3.0%), *P* = 0.040 (grade B 4.3%, grade C 4.3% vs grade B 5.6%, grade C 0.0%). Operative time of the HJ decreased along the maturation of experience, that is, it showed a significantly negative correlation with the case number: HJ time overall Rho −0.144 (95% CI −0.282 to −0.036), and HJ suturing time −0.167 (95% CI −0.287 to −0.041). For more details, see Appendix 2 (Supplemental Digital Content 1, http://links.lww.com/SLA/F413). Grade B/C BL was positively associated with POPF grade B/C (11.5% vs 5.6%, OR: 2.185, *P* = 0.085). However, after excluding concomitant POPF cases, the total OSATS score remained a predictor of grade B/C BL (R: 0.481, *P* = 0.016).

**FIGURE 3:**
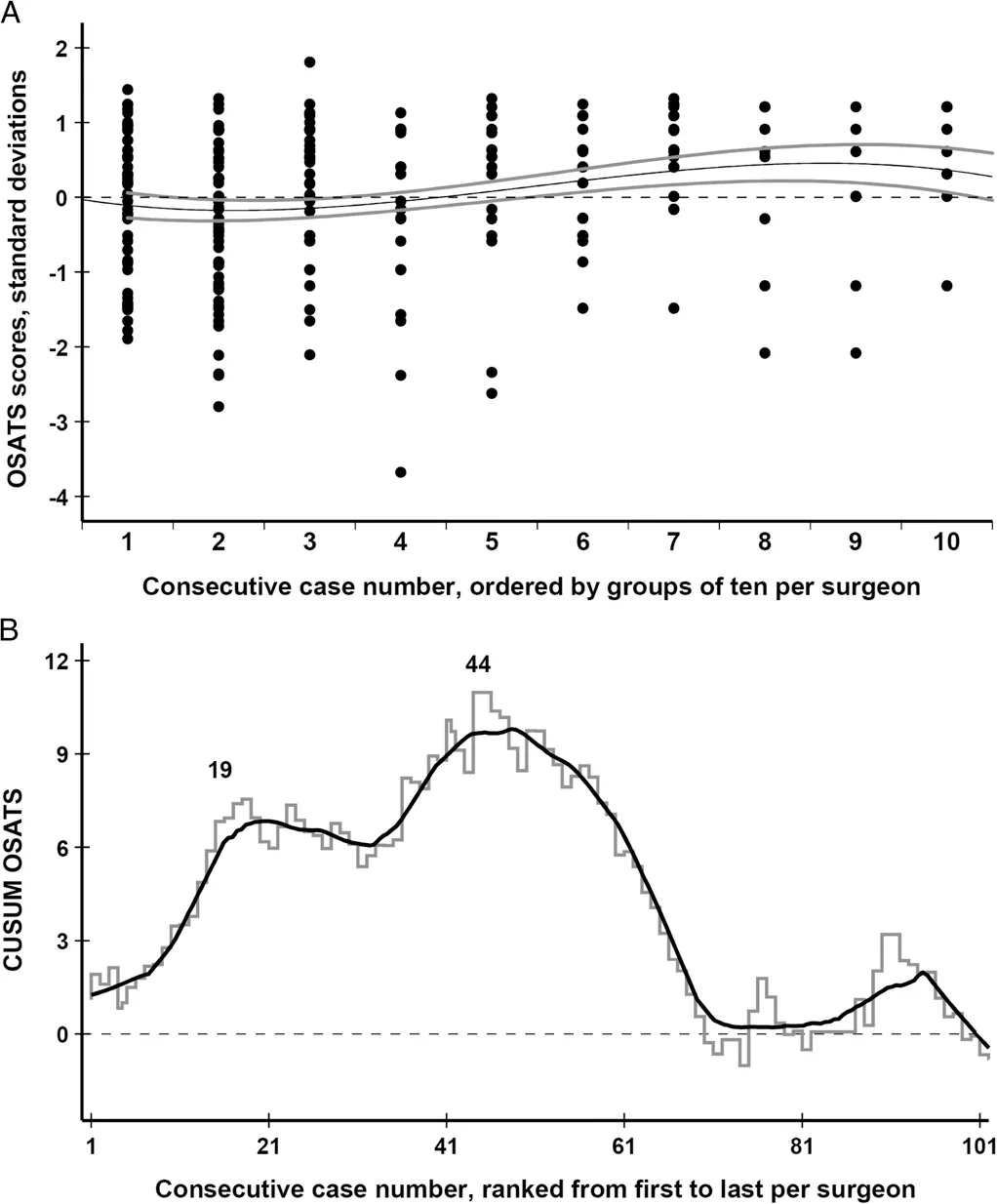
OSATS learning curve of robotic HJ in CUSUM analysis. A, A significant association with consecutive case number was observed at 0.270 (95% CI: 0.153–0.380). B, Stabilization of the CUSUM learning curve was reached at 19 robotic HJ procedures, and after 44 procedures, the learning curve showed a continuous downward trend. The CUSUM curve represents the aggregate of individual learning curves of surgeons in the study, with each surgeon’s consecutive case number contributing to the total. The average number of RPDs per surgeon was 40.7, but some surgeons performed significantly more procedures, thus extending the CUSUM curve beyond the average.

### Effect of Previous Experience on Objective Structured Assessment of Technical Skills and Intergrader Reliability

Surgeon characteristics had a significant influence on OSATS scores. Median OSATS scores differed significantly for several variables (for more information, see Table 4). In multivariable analysis, OSATS scores were significantly influenced by the cumulative number of HJs performed (standardized coefficient beta = 0.273, *t* = 3.303, *P* < 0.001), and prior experience in laparoscopic PD (standardized coefficient beta = −0.145, *t* = 1.996, *P* = 0.047).

**TABLE 4:** Surgeon Characteristics Affecting OSATS

	Univariable		Multivariable		
Surgeon characteristic	Rho	*P*	SCB	*T*	*P*
University medical center	0.287	<0.001	0.130	0.841	0.401
Attending surgeon	−0.236	<0.001	−0.215	−1.739	0.083
Increasing surgical experience (yr)	−0.160	0.010	−0.001	−0.005	0.996
Increasing MI surgical experience (yr)	−0.229	<0.001	0.082	0.719	0.473
Surgeon annual volume >20 RPDs	0.280	<0.001	0.071	0.418	0.676
Prior experience in laparoscopic PD	−0.143	0.022	−0.145	−1.996	0.047
Cumulative experience in robotic HJ	0.293	<0.001	0.273	3.858	<0.001

SCB indicates standardized coefficient beta.

Since prior experience in laparoscopic PD affected OSATS, learning curves were also analyzed separately. In surgeons with prior experience in laparoscopic PD, a gradual decline in the learning curve was initiated at 19 procedures. This was different from surgeons without prior experience in laparoscopic PD, as the learning curve showed a plateau at 19 procedures, where the decline started after 44 procedures (Appendix 3, Supplemental Digital Content 1, http://links.lww.com/SLA/F413). In addition, OSATS remained a consistent risk factor for B/C BL in a sensitivity analysis excluding fellows (68/259 HJs; OR: 0.283, 95% CI: 0.082 to 0.976, *P* = 0.046), whereas surgeon characteristics showed no correlation (academic center, increasing surgical experience, increasing MI surgical experience, surgeon annual volume, prior experience in LPD).

The validation grader validated a random sample of 65 HJs. Intergrader variability analysis showed a positive correlation (Rho: 0.255, *P* = 0.044). All elements of OSATS demonstrated a positive correlation between graders: gentleness (Rho: 0.066, *P* = 0.606), time and motion (Rho: 0.226, *P* = 0.075), instrument handling (Rho: 0.193, *P* = 0.129), flow of operation (Rho: 0.277, *P* = 0.028), tissue exposure (Rho: 0.200, *P* = 0.116), and overall technical skill (Rho: 0.208, *P* = 0.101).

## DISCUSSION

This first multicenter study to assess the impact of surgical performance during robotic HJ in RPD found that an OSATS score>21 (attainable scores: 6 to 30) is associated with a ~60% relative reduction of grade B/C BL (5.1% vs 12.5%, *P* = 0.013). This reduction remained consistent in multivariable analysis including baseline variables, HJ characteristics, mitigating strategies, surgical, and surgical experience. The OSATS learning curve stabilized after 19 RPD procedures, along with a significant increase in OSATS (17.7 vs 26.0*, P* < 0.001), and a significantly lower rate of grade B/C BL (8.6% vs 5.6%, *P* = 0.040). The total experience with robotic HJ in RPD had the highest impact on OSATS, whereas previous experience in laparoscopic PD demonstrated a significant impact on shortening the learning curve.

The OSATS score has frequently been used to assess surgical performance and the learning curve in laparoscopic surgery.^17,21–24^ For example, in the LEOPARD-2 trial laparoscopic pancreatico-jejunostomy was assessed.^25^ Although this study included surgeons with at least 20 LPDs in the LAELAPS-2 program,^11^ a low OSATS was found (22% received a technical summary score of below average—a score below 3 out of 5).^21^ In the present study, grade B/C BL occurred in 18 out of 259 patients (6.9%). This rate is lower than reported in the 4 randomized trials on laparoscopic versus open PD (laparoscopic arm 11.9% vs 12.8% in the open PD arm).^25–28^ The question now is whether the BL occurred because of technical difficulties (ie, “difficult HJ”) or suboptimal surgical performance. In the current study, bile duct diameter was similar in the low-scoring and high-scoring groups of OSATS scores [median: 10 mm (7 to 15) vs median: 10 mm (7 to 15), *P* = 0.385). In addition, bile duct aspect, additional segments anastomosed, and distance to hilar plate were similar between both groups. Finally, there was no significant correlation between bile duct diameter and grade B/C bile leakage. In conclusion, in the current study, the difficulty of the HJ was not associated with OSATS scores or bile leakage.

Although multiple studies demonstrated a significant association of OSATS with outcomes in laparoscopic surgery,^13^ studies on surgical performance assessment using OSATS for robotic surgery are rare.^29^ In a single center study, OSATS was assessed for the robotic HJ on biotissue,^30^ where expert surgeons reached a median OSATS of 29 (out of 30). Accordingly, in 2 large single-center studies, the rates of BL among expert surgeons ranged from 1/132 in RPD (0.8%) to 28/2000 (1.4%) in OPD.^31,32^ Giulianotti et al reported a low (2.6%) BL rate after robotic HJ, and identified independent risk factors.^33^ The latter resonates with the current study, suggesting that the rate of BL can be lowered with further improvement in OSATS scores. However, the graders incorporate components not represented in the OSATS score more critically in the summary score, e.g., optimal distance between sutures. Currently, evidence on the type of HJ with the lowest BL rate is lacking. Future research should investigate the effect of independent risk factors, OSATS scores and technical factors in the prediction of BL. This could validate the assessment to determine the optimal technique for the HJ.

The learning curve is associated with a negative impact on patient outcomes. This is reflected by a meta-analysis of learning curves for pancreatic surgery, in which the initial phase of the learning curve for laparoscopic PD was 39 procedures and associated with a significant decrease in complications.^34^ In the LAELAPS-3 cohort, analysis of the surgeons’ learning curves for RPD demonstrated a turning point for feasibility at 15 procedures and proficiency at 62 procedures. This is comparable to a previous study^35^ where the learning curve inflection point of pancreatic anastomosis was reached after 40 procedures (27.5% vs 14.4%; *P* = 0.04). Robot HJ during choledochal cyst excision (N = 60) showed a learning curve of 14 procedures.^36^ The pancreatic anastomosis learning curve was reflected by the LAELAPS-3 cohort (33 PJs),^15^ and resonates with the learning curve of the HJ in the current study: plateau at 19 procedures, completion at 44 procedures.

With continued experience in addition to a structured training program, outcomes of RPD will improve, resulting in fewer complications, shorter operative time, and reduced blood loss.^18,37^ To improve surgical skills in robotic surgical procedures, all surgeons participating in our study completed a structured training program in RPD. This method,^38^ first developed at the UPMC, has been shown to significantly increase the OSATS score.^30^ The OSATS cutoff of 21 points could serve as a proficiency performance bar before RPDs; however, such a score for HJs on artificial organs would have a high impact on standards of surgical education and require a rigorous validated multicenter cohort. The current OSATS scoring system is time-consuming and not feasible for real-time assessment during surgery. However, we believe that OSATS remains a valuable tool for surgical education and training, as it allows for structured and objective feedback on surgical performance. The time-consuming nature of OSATS evaluation does pose challenges for scalability in training. However, we believe that future developments in automated video analysis and machine learning will offer solutions to this issue. Automated systems will be able to analyze surgical videos and provide real-time feedback on technical performance, making (OSATS) assessment more efficient and scalable. In the meantime, we believe that OSATS can still be effectively used in training programs through postoperative video review and feedback sessions.

Several limitations of this study should be taken into account. First, we cannot exclude that some BLs in our cohort were actually related to POPF. However, since the rate of BL in our cohort is low compared with previous studies, we consider this less likely to be a relevant bias. Furthermore, OSATS remained predictive when excluding POPF patients. Second, the rate of BL in this cohort (6.9%) was slightly lower than previously reported in the entire LAELAPS-3 cohort (8%),^19^ which could indicate some chronological bias. Third, our findings are based on specifically trained surgeons. This may impact the learning curve reported in this study. Strengths of this study include its large cohort size of surgeons at the beginning of the learning curve. In addition, all participating surgeons completed a specialized training program (LAELAPS-3) based on the UPMC training model.^38^

## CONCLUSIONS

OSATS scoring of surgeon’s performance during robotic HJ in RPD identified a reduced risk of clinically relevant BL during the maturation of experience. OSATS cutoff based on video grading requires further research, as it may provide a performance bar for competency-based training and thus facilitate the safe introduction of RPD. These findings underline the importance of surgical proficiency through training in preventing postoperative complications.

## Supplementary Material
