# Supplementary material for: Video Grading of Bile Duct Anastomoses During Robotic Pancreatoduodenectomy Predicts Bile Leak and Identifies the Learning Curve: A Multicenter Study
Source: Ann Surg. 2025 Feb 17;284(6):1102–10. doi: 10.1097/SLA.0000000000006673 (PMC13764248; doi:10.1097/SLA.0000000000006673)
Supplement: Supplementary file 1 [file sla-284-1102-s001.docx]

## APPENDIX 1 Title: Threshold analysis with the risk ratio for bile leakage per OSATS score

|  |  | **Univariable** | |  |  | **Multivariable** |  | |  |
| --- | --- | --- | --- | --- | --- | --- | --- | --- | --- |
| **OSATS cut-off** | **Odds (95%CI)** | | **P value** | | **Odds (95%CI)** | | | | |
| 14 | 0.187 (0.039−0.896) | | <.001 | | 0.126 (0.016−1.015) | | | | |
| 15 | 0.261 (0.059−1.168) | | <.001 | | 0.166 (0.023−1.228) | | | | |
| 16 | 0.485 (0.119−1.988) | | .003 | | 0.358 (0.063−2.046) | | | | |
| 17 | 0.573 (0.190−1.727) | | <.001 | | 0.540 (0.132−2.209) | | | | |
| 18 | 0.478 (0.212−1.076) | | <.001 | | 0.339 (0.102−1.126) | | | | |
| 19 | 0.523 (0.257−1.002) | | .002 | | 0.328 (0.104−1.037) | | | | |
| 20 | 0.566 (0.302−1.058) | | .010 | | 0.347 (0.111−1.080) | | | | |
| 21 | 0.523 (0.297−0.920) | | .013 | | 0.273 (0.088−0.849) | | | | |
| 22 | 0.663 (0.382−1.151) | | .052 | | 0.432 (0.145−1.289) | | | | |
| 23 | 0.714 (0.436−1.167) | | .125 | | 0.515 (0.181−1.462) | | | | |
| 24 | 0.747 (0.504−1.107) | | .272 | | 0.455 (0.153−1.353) | | | | |
| 25 | 0.869 (0.590−1.280) | | .491 | | 0.609 (0.204−1.820) | | | | |
| 26 | 0.919 (0.681−1.241) | | .264 | | 0.606 (0.188−1.953) | | | | |
| 27 | 0.956 (0.770−1.187) | | .512 | | 0.723 (0.189−2.768) | | | | |
| 28 | 1.066 (0.863−1.316) | | .306 | | 1.486 (0.354−6.239) | | | | |
| 29 | * | | .625 | | * | | | | |
| P value, based on Chi-Square for Grade B and C. *zero BL.  Odds ratios are typically used for binary variables/categories. In this special case however, they may not fully reflect the ordinal nature of bile leakage. We have chosen to report the p-values based on the Chi-squared test, which accounts for the ordinal categories and provides a more accurate assessment of statistical significance. | | | | | | | |  |  |

## APPENDIX 2

**Title**: Operative time learning curve of robotic hepaticojejunostomy

**Rho = -0.144, *P* =0.010**

## APPENDIX 3

**Title**: The OSATS learning curve of robotic hepaticojejunostomy in cumulative sum analysis for surgeons with- and without experience in laparoscopic pancreatoduodenectomy
